# Robotic surgery for deep-infiltrating endometriosis: is it time to take a step forward?

**DOI:** 10.3389/fmed.2024.1387036

**Published:** 2024-03-05

**Authors:** Filippo Alberto Ferrari, Youssef Youssef, Antoine Naem, Federico Ferrari, Franco Odicino, Harald Krentel, Gaby Moawad

**Affiliations:** ^1^Department of Obstetrics and Gynaecology, AOUI Verona, University of Verona, Verona, Italy; ^2^Division of Minimally Invasive Gynecologic Surgery, Department of Obstetrics and Gynaecology-Maimonides Medical Center, Brooklyn, NY, United States; ^3^Faculty of Mathematics and Computer Science, University of Bremen, Bremen, Germany; ^4^Department of Obstetrics, Gynecology, Gynecologic Oncology, and Senology, Bethesda Hospital Duisburg, Duisburg, Germany; ^5^Department of Clinical and Experimental Sciences, Division of Obstetrics and Gynecology, University of Brescia, Brescia, Italy; ^6^Department of Obstetrics and Gynecology, George Washington University, Washington, DC, United States; ^7^The Center for Endometriosis and Advanced Pelvic Surgery, Washington, DC, United States

**Keywords:** endometriosis, robotic surgery, laparoscopy, diaphragm, urinary tract, Colon, rectum

## Abstract

Endometriosis is a chronic debilitating disease that affects nearly 10% of women of the reproductive age. Although the treatment modalities of endometriosis are numerous, surgical excision of the endometriotic implants and nodules remains the sole cytoreductive approach. Laparoscopic excision of endometriosis was proven to be beneficial in improving the postoperative pain and fertility. Moreover, it was also proved to be safe and efficient in treating the visceral localization of deep endometriosis, such as urinary and colorectal endometriosis. More recently, robotic-assisted surgery gained attention in the field of endometriosis surgery. Although the robotic technology provides a 3D vision of the surgical field and 7-degree of freedom motion, the safety, efficacy, and cost-effectiveness of this approach are yet to be determined. With this paper, we aim to review the available evidence regarding the role of robotic surgery in the management of endometriosis along with the current practices in the field.

## 1 Introduction

Endometriosis is one of the most common gynecologic diseases affecting nearly 10% of women of the reproductive age (1). Endometriosis is defined by the presence of endometrial-like glands and/or stroma out of the uterus (2, 3). The clinical manifestations of endometriosis could be broadly categorized into endometriosis-associated pain and infertility (4). The most commonly-reported symptoms of endometriosis are chronic pelvic pain, dysmenorrhea, and dyspareunia (5). On the other hand, infertility is reported to affect 30–50% of endometriosis patients (6). Although endometriosis has various forms and manifestations, superficial peritoneal endometriosis, ovarian endometriomas, and deep endometriosis are the three main types of the disease (7). Deep endometriosis has been historically defined as deep infiltrating endometriosis extending 5 mm below the peritoneal surface (8). However, a recent international terminology consensus has argued that measuring depth in millimeters is inaccurate. It is now agreed that any endometrial-like tissue in the abdomen, extending on or under the peritoneal surface, is referred to as deep endometriosis (9). These lesions are typically nodular, capable of invading adjacent structures, and associated with fibrosis, leading to the disruption of the normal anatomy (9). Such lesions usually involve the retro-cervical space, the recto-vaginal septum, the uterosacral ligaments, as well as nearby organs such as the sigmoid colon, rectum, bladder, and ureters (10, 11). It should be noted that bowel endometriosis is a special subtype of deep endometriosis that should be only diagnosed when the muscular layer of the bowel wall is infiltrated with the disease (12, 13). Hormonal suppressive treatments with cyclic oral contraceptive pills, progestins, and gonadotropin-releasing hormones (GnRH) agonists and antagonists were proven to be safe and effective in treating the endometriosis-associated pain (14–16). However, those therapies are suppressive rather than cytoreductive, which means, in most cases, the symptoms recur with the suspension of the treatment. This becomes particularly problematic in cases of infertility or when the patient seeks conception. To date, surgical excision of endometriosis is the only cytoreductive approach with promising symptom-relief rates. Furthermore, surgery becomes unavoidable when organ damage is suspected or already detected (4, 17). The basic principles of the endometriosis excisional surgery are the uncomplicated resection of the visualized endometriotic lesions, performing adhesiolysis, and restoring the normal pelvic anatomy (18). Minimally invasive surgery (MIS) is actually the approach of choice since it demonstrated reduced blood loss, postoperative pain, and duration of hospitalization. In fact, the Enhanced Recovery After Surgery (ERAS) program recommends MIS to improve the postoperative patient recovery (19, 20). Nevertheless, the laparoscopic management of advanced and complex cases is challenging due to tissue alterations provoked by adhesions and the endometriosis-associated fibrosis (21). Despite the advantages of laparoscopy compared to open surgery and the development of laparoscopic 3D optics, the laparoscopic approach harbors technical limitations in terms of ergonomics and the limited range of motion (22). Robotic-assisted surgery was developed more than 30 years ago as a United States military project and received the approval of the Food and Drug Administration (FDA) in 2005 (23). Since then, robotic-assisted surgery has been widely implemented and adopted in gynecology (24). Robotic-assisted surgery was recently reported to have shorter operation time and less blood loss than laparoscopic surgery, with comparable outcomes (25). However, the available data in that regard is conflicting and more studies are required to justify this claim. Robotic-assisted surgery with its rapidly evolving technology can overcome much of the laparoscopic limitations, and represents a step forward toward a safer and more precise excision of the disease. Indeed, the EndoWrist^®^ increases the range of motion and the robotic platform 3D vision avoids the problem of an unstable bi-dimensional image totally dependent on the assistant. Nonetheless, its superiority or at least non-inferiority in the management of deep endometriosis remains unclear due to the lack of research in the field. The present review aims to provide an update on the role of robotic-assisted surgery in managing endometriosis and summarize the main scientific findings in the literature.

## 2 Materials and methods

This work is a narrative review of the role of robotic-assisted surgery in deep-infiltrating endometriosis. A broad scope search of literature was conducted in Scopus, PubMed/Medline, ScienceDirect and the Cochrane Library. A combination of the following keywords was used: deep-infiltrating endometriosis, robotic surgery, robot-assisted laparoscopy. The search was restricted to only include articles in English language. Relevant papers of all types (i.e., original articles, video articles, and case reports) were assessed and included as appropriate.

## 3 Feasibility of the robotic-assisted surgery

Laparoscopic excisional surgery is the gold standard for the treatment of deep endometriosis. More recently, robotic-assisted surgery became more frequently adopted for the surgical management of endometriosis without clear indications. Available non-comparative studies of women that were operated robotically found a comparable complication rate between robotic-assisted surgery and laparoscopy with a significant reduction of pain symptoms. An improved quality of life at follow-up was also reported (22, 26–32). Nonetheless, very few studies that compared the two minimally invasive approaches in patients with r-ASRM stage III/IV endometriosis are available. To the best of our knowledge, there are only one randomized-controlled trial (RCT) (33) and two meta-analyses (34, 35) in that regard.

In 2010, Nezhat et al. (36) published for the first time a retrospective study comparing robotic-assisted surgery and laparoscopy in severe endometriosis. Although the outcomes and complication rates were comparable between the two groups, longer operative time and hospital stay were noted in the robotic group. The mean difference in the operation times was 61 min.

The safety and feasibility of robotic-assisted surgery was further confirmed by several studies that reported comparable outcomes and rates of intra-and postoperative complications (37–41).

In a large retrospective study by Nezhat et al. (41), the hospital stay was longer in the robotic-assisted group in contrast to the findings of other reports. In that study, only 23% of patients in the laparoscopy arm stayed overnight in the hospital against all the patients of the robotic arm without any complication in both groups (41). In our opinion, these findings may be related to a standardized protocol of postoperative discharge rather than an actual underlying difference between both approaches.

The total operative time was significantly shorter in the laparoscopy group in the majority of the studies (38, 40–42). Dulemba et al. (37) reported a non-significant difference in the length of surgery in accordance the multivariate analysis of Magrina et al. (39), which accounts for the impact of the higher number of procedures and radicality in the robotic group (39). In the same study, the authors reported a higher rate of histological confirmation of endometriosis in the robotic-assisted surgery group compared to the laparoscopic counterpart (80% vs. 56.8%, respectively). This could be attributed to the technology of the robotic platform and its three-dimensional visualization. Improved visualization could logically lead to improved detection of superficial lesions, which is of paramount importance in women reporting pelvic pain suggestive for endometriosis.

For some authors, obesity is a major limiting factor for laparoscopic surgery in terms of some technical aspects and the difficulties to access to the surgical spaces (40). Nonetheless, the available evidence supported the feasibility and safety of robotic-assisted gynecologic surgery in obese patients (43–46). In recent years, the wide spread of robotic platforms increased the number of women treated with minimally invasive approach (47, 48). Nezhat et al. (40) speculated that obese patients may benefit from robotic-assisted surgery more than normal-weighted patients. However, their study reported comparable outcomes and a significant higher total operative time in the robotic-assisted surgery arm compared to laparoscopic arm in the obese subgroup (40). Other authors addressed the increased amount of time to the multiple changing in table positioning but the proposal of a hybrid robotic-laparoscopic procedure was not demonstrated to be a time-saving option (38).

In 2017, Soto et al. (33) published a randomized controlled trial (LAROSE trial) enrolling 73 patients randomly assigned to laparoscopy or robotic-assisted surgery (33). To the best of our knowledge, this is the only trial available to date in that regard. Multivariate analysis showed no significant differences in total operative time, intraoperative complications and blood loss between the two groups. Nonetheless, only 33% of the patients had stage III/IV endometriosis and the intraoperative staging was significantly lower in the robotic arm. When taking in consideration the low rate of complications and adverse outcomes as well as the small sample size, the conclusion of the study may not be generalizable.

In a recent meta-analysis by Restaino et al. (35), the safety of robotic-assisted surgery was confirmed with a comparable rate of intra-and post-operative complications. In addition, the authors reported similar estimated blood loss quantities between the two groups. Moreover, robotic-assisted surgery was associated with longer operative time compared to laparoscopic surgery, even when excluding the docking time (35). Nonetheless, the authors concluded that the heterogeneity in outcomes of the included studies and the focus on the peri-operative window did not allow any conclusions on long-term pain relief, quality of life and fertility results (34, 35). Moreover, some of the considered studies enrolled both mild and severe endometriosis (33, 41) while other authors failed to report the stage of the disease, which contributed to the wide heterogeneity in the included population. Those results are in accordance with the results Chen et al. (34).

## 4 Colorectal endometriosis

Bowel endometriosis is a subgroup of deep endometriosis that involves the recto-sigmoid junction in the majority of the cases (65%), followed by the rectum (15–20%) (40, 49). In the available literature, its incidence was reported to be 4–38% in women with endometriosis and cyclic bowel symptoms, especially dyschezia and hematochezia (50). The surgical management is required after failure of conservative medical therapies and it should be tailored on the patient’s symptoms and disease characteristics. Although clear guidelines are lacking, the choice between segmental resection with anastomosis, discoid resection or nodulectomy (shaving) is mainly based on the size, the depth of the lesions’ invasion, the circumference of the disease and the coexistence of skip lesions (29, 51). In the last years, some authors considered the robotic-assisted surgery in cases of bowel endometriosis to overcome the complexity and technical difficulty of advanced stages allowing a smoother preparation of the rectum with an easier superior rectal artery sparing and simpler handling of the tissue during the anastomosis (23, 29). In a meta-analysis of a total of 3,079 women with recto-sigmoidal endometriosis, the statistical analysis demonstrated a higher rate of major complications for segmental resection (11.8%), followed by discoid resections (7.5%) and the rectal shaving technique (5.5%). In 92% of cases, a minimally invasive approach was used but robotic-assisted surgery was performed only in 1.7% of the patients (49).

In 2011, Nezhat et al. published two successful cases of bowel endometriosis managed with robotic segmental rectal resection and discoid resection demonstrating the feasibility of both approaches (28). In a small comparative study, Lim et al. (52) compared robotic-assisted anterior rectal resection with the open approach. The authors failed to detect any significant differences in total operative time, blood loss and length of hospitalization. A higher number of complications was reported in the laparotomy group, but the difference was not significant (52). In a cohort of 22 consecutive patients, robotic-assisted excision of bowel endometriosis was confirmed to be safe and feasible, with satisfactory short-term results and zero conversions to laparotomy (26).

In a recent prospective cohort study, the comparison between laparoscopy and robotic-assisted surgery did not yield in any differences in blood loss, intra-operative and postoperative complications, and voiding dysfunction rates. The robotic arm had a longer total operative time (221 ± 94 min vs. 163 ± 83 min, *p* = 0.03), a longer hospital stay (8 ± 4.4 vs. 6.5 ± 2.6 days, *p* = 0.18), a higher number of grade III complications (according to Clavien Dindo Classification) without reaching the statistical significance (53).

Raimondo et al. (54) published the results of a multicentric prospective cohort study comparing laparoscopy with robotic-assisted surgery. The data of the 44 enrolled women showed no differences in outcomes, complications, operative time (skin to skin) and improvement of symptoms at 12 months of follow-up. A longer operative room time in the robotic arm was reported (296 ± 80 min vs. 241 ± 72 min; *p* = 0.020). This is also consistent with the findings of Ercoli et al. (26).

## 5 Diaphragmatic endometriosis

Diaphragmatic endometriosis is a rare form of the disease. The exact incidence and prevalence of diaphragmatic endometriosis are unknown precisely yet. However, the prevalence of diaphragmatic endometriosis was reported to be 1.86–4.7% (55). The preoperative diagnosis is difficult and the management remains controversial (45, 46, 55, 56). It may cause catamenial symptoms or chronic pain. Nonetheless, some cases may be asymptomatic (57, 58).

Ceccaroni et al. reported the portion of the diaphragm behind the right hepatic lobe as the most frequent localization (57). Redwine (56) postulated the existence of sentinel lesion on the anterior part of the diaphragmatic peritoneum which could suggest the presence of more extended localization and may induce the surgeon to a complete retro-hepatic exploration (56). Symptomatic lesions are associated with a deep involvement of the whole thickness of the diaphragm and an association with symptomatic pelvic or bowel disease was reported in the totality of the cases (59, 60).

The management is a real challenge in particular for the rarity of the localization, availability of few case series, lack of guidelines and difficulty in the preoperative diagnosis. Complete surgical resection avoiding the opening of the thoracic cavity is the goal if a full-thickness excision is not required (61). Laparotomic, laparoscopic and robotic approaches were reported in the literature, associated with video-assisted thoracoscopy (VATS) when thoracic symptoms were present (27, 57, 60, 62). Thermal ablation was proposed for the superficial lesions and Ceccaroni et al. favored Argon Beam Coagulator (ABC) than electrocautery (57).

Abo et al. published a case series of 35 patients in which robotic-assisted endometriosis excision was performed over a period of 30 months (27). Among them 8 cases of diaphragmatic localization were reported. No major complications were related to the procedure but the extent of the disease and surgical technique was not described. Recently, Roman et al. published a proposal to standardize the surgical management using robotic surgery reporting the feasibility, safety and reproducibility of this approach (62). Moreover, cases of incidental tension pneumothorax during inspection of the abdomen in patients treated with robotic-assisted surgery is reported and the entire surgical team needs to be aware of this possibility (60, 63).

However, it should be noted that in a recent study of Naem et al. (55) patients with diaphragmatic endometriosis were followed up for a mean duration of 23 months. Although 78.9% of patients reported major postoperative improvement, the postoperative recurrence rates of diaphragmatic endometriosis-related symptoms were higher than expected, with complete pain relief being reported in 25–50% of patients. On the other hand, asymptomatic lesions that were left *in situ* remained asymptomatic after a follow up period of 6–14 months (55). Therefore, caution should be made before operating diaphragmatic endometriosis, especially in the asymptomatic cases, where treatment seems to be unnecessary, and appropriate patient counseling about what to exactly expect postoperatively should be carried out (55, 64).

## 6 Urinary endometriosis

The urinary tract is rarely an endometriosis localization occurring in 0.5 to 12% of women with pelvic endometriosis. The prevalence exceeds 50% in patients with deep endometriosis (65, 66). The urinary bladder is the most common site (80%), followed by the ureter (15%), kidney (3%) and urethra (2%) (65, 67). The definition and incidence of bladder endometriosis are different in the literature owing to the variation in the inclusion or exclusion of superficial serosal lesions. Related symptoms frequently include dysuria, hematuria, suprapubic pain and urinary frequency (65). Ureteral endometriosis is less frequent and most commonly affects the left distal ureter (68). It can be classified in extrinsic form when the ureter is involved by an external nodule and intrinsic form if mural invasion is present (68). The symptoms related to ureteral endometriosis may be lower back pain, recurrent urinary tract infections, and hematuria. However, it remains asymptomatic in around 50% of the cases and may lead to an ipsilateral silent kidney (65). When surgery is required, minimally invasive approaches were demonstrated to provide adequate outcomes and acceptable rate of complications in case of urinary tract endometriosis (67, 69). In case of bladder endometriosis, the majority of the authors suggested to perform partial cystectomy to achieve a complete resection of the nodule (65, 67, 68). According to literature, ureteral lesions may be removed with ureterolysis, segmental excision with end-to-end anastomosis or reimplantation (65, 67, 69).

In the literature there are no randomized trials or prospective studies comparing laparotomy with laparoscopy and robotic-assisted surgery in case of urinary tract endometriosis. However, case reports and case series demonstrated the feasibility and safety of the robotic-assisted laparoscopy (22, 27, 29, 65, 66, 68, 70–72). A French multicenter retrospective cohort including 232 patients reported the use of robotic surgery in 14.7% of the patients in comparison to laparoscopy and laparotomy in 74.1 and 11.2% of cases, respectively (68). Di Maida et al. (66) published a series of 74 women underwent minimally invasive surgery for urinary tract endometriosis. Twenty-eight (37.8%) were managed with laparoscopy and 46 (62.2%) with robotic-assisted surgery. The authors demonstrated the feasibility of the approach and reported an overall postoperative complication rate of 10.9% in the robotic group, which is consistent with the findings of Giannini et al. (70). A retrospective study compared laparoscopy and robotic-assisted surgery for the treatment of bladder endometriosis with partial cystectomy. No differences in term of surgical outcomes, perioperative complications, blood loss and recurrence rates were observed.

## 7 Sacral plexus endometriosis

Deep endometriosis involving the sacral plexus and the large nerves of the pelvis is deemed to be rare in gynecology (73). Although the first report of deep endometriosis of the sciatic nerve dates back to 1955 (74), very few data are available regarding its precise prevalence and optimal management. This may be attributed to the lack of awareness of this condition due to the lack of correlation between endometriosis, menstruation, and the resulting neurological symptoms (75). Deep endometriosis may involve the pelvic neural structures mainly in two ways. The first and most common form of neural involvement includes compressing the sciatic nerve and sacral roots due to the posterolateral extension of parametrial and rectovaginal endometriosis, causing intrapelvic nerve entrapment (76). It is noteworthy that rectovaginal nodules tend to involve the sacral roots S2, S3, and S4. While deep nodules of the parametrium with more superior lateral localization tend to involve the sciatic nerve (76). The second form of involvement is the direct infiltration of the nerves with endometriosis. This form is less common and was reported to account for nearly 33.5% of patients with recurrent sciatica (77). Pelvic nerve involvement with endometriosis causes a variety of somatic sensory and motor symptoms, with or without pelvic organ dysfunction (78, 79). In cases of sciatic nerve involvement, the patients often report cyclic sciatica. The term sciatica refers to pain along the distribution of sciatic nerve, usually referring to leg and gluteal pain (79). In addition, foot drop and alteration in the Achill’s tendon reflex may be noticed (76). On the other hand, when the sacral roots are involved with endometriosis the patients suffer from perineal pain, altered sensations in the dermatomes S2 to S4, and pelvic organ dysfunction, such as constipation, vaginal dryness, urinary urgency or bladder atonia (76, 78). It should be noted that such symptoms do not necessarily originate from the sole involvement of the sacral roots, but the involvement of the hypogastric nerves, splanchnic nerves, and inferior hypogastric plexus in the large rectovaginal or parametrial endometriotic nodule (76).

Deep endometriosis involving the sacral roots and sciatic nerves was historically treated with laparoscopic detrapment of the involved structures in the means of neural decompression and shaving at the epineurium level. Less commonly, partial nerve resection was also applied (76–78, 80). Furthermore, laparoscopic identification and subsequent excision of peritoneal pockets resulted also in a postoperative resolution of the neurologic pain symptoms (79). The efficacy of such interventions is not estimated precisely yet, but the available reports indicate that pain symptoms tend to be improved postoperatively (76, 80). It should be noted that postoperative bladder dysfunction and the need for self-catheterization was recorded in 5.8% of the operated patients in the series of Roman et al. (76) over a year of follow-up. In the same series, the authors reported that *de novo* hyperesthesia, hypoesthesia, or allodynia were recorded in 17.2% of patients postoperatively (76).

On this basis, the role of the robotic-assisted surgery, which is basically a subdivision of laparoscopic surgery, is far from being determined. Available reports indicate that robotic surgery with its 3D image and the 7-degree of freedom of the robotic instruments increase the safety and the precision of the neural dissection (81, 82). Other authors used indocyanine green during robotic-assisted surgery for deep endometriosis to examine the vascularization of the hypogastric nerves and inferior hypogastric plexus, and subsequently their viability (83). To date, there are no studies comparing the operative and postoperative outcomes of robotic-assisted surgery compared to laparoscopy in terms of operative time, blood loss, short-and long-term postoperative neurologic symptoms.

## 8 Discussion

The available literature indicates the feasibility and safety of robotic-assisted surgery in treating deep endometriosis. However, drawing definitive conclusions regarding its superiority or non-inferiority for patients with advanced endometriosis is challenging due to several factors. These include the limited number of studies, their heterogeneity, and the predominance of retrospective designs. Additionally, comprehensive investigations into crucial long-term outcomes such as sustained pain relief, variations in quality of life, and fertility outcomes have been infrequent or inadequately conducted.

Nowadays, minimally invasive approaches are considered the gold standard for the surgical treatment of deep endometriosis and in this setting the robotic-assisted surgery may provide the technology to overcome some of the limitations of laparoscopy allowing a more ergonomic position, three dimensional vision and freedom of wrist movement (40). Some surgeons use robotic-assisted surgery in deep endometriosis claiming an advantage in complex pelvic pathology, obese patients and prior surgical history. However, such studies may be subject to selection bias (23, 29). Several studies demonstrated that the two main limitations to the spread of robotic-assisted surgery are longer operative time and higher costs (23). The increased total operative time is related partially to the phase of docking and intuitively to the specific learning curve of robotic-assisted surgery (84). Moreover, some authors underlined the need for changing the table position and hybrid conventional/robot-assisted laparoscopy in advanced procedures in consideration of the arm maneuverability in the extrapelvic surgical field and absence of interchangeability of the camera between ports (29, 41). Finally, robotic-assisted surgery lacks the tactile feedback and seemed to correlate with longer operative time, making the tissue dissection more difficult and the identification of the lesions limited (85). It should be noted that DaVinci (Intuitive Surgical, United States) has been the main surgical robot used by different surgical specialties worldwide. More recently, the Hugo^™^ RAS system (Medtronic, Minneapolis, United States) was introduced and implicated in the management of deep endometriosis (86). The initial experience with this new system indicated its safety in terms of perioperative complications and efficiency in terms of postoperative symptom relief (87, 88). However, the median docking time in one series could be considered long in comparison with the docking time required for DaVinci (87). This could be attributed to the learning curve, since this system is still new and the surgeons may not be very experienced, or due to the multiple bedside arms that should be brought to the operation theater and ducked.

Robotics surgery lead to substantial additional costs compared to laparoscopy, not only for the operative time but also the need of staff training, licenses and maintenance (89). However, recently a trend in cost reduction was registered due to shorter hospital stay, operative time and better resources’ administration compared to initial experiences (90). These findings may suggest avoiding the overestimation of the costs drawback of robotic-assisted surgery and to run studies of suitable design investigating the economic impact in well trained and dedicated team.

One of the major complications of deep endometriosis treatment is the postoperative onset of sexual, rectal and voiding dysfunction that may affected more than 50% of the women (29, 91). Different expert groups described standardized approaches of nerve sparing with a systematic identification of the hypogastric nerves, pelvic splanchnic nerves and pelvic plexus in order to reduce denervation (18, 92, 93). Nonetheless, the preservation of the pelvic autonomic nerves requires not only excellent knowledge of pelvic anatomy, but also great laparoscopic technical skills (92). In this setting, all the latest technical development brought by robotic-assisted surgery may be considered especially helpful to increase the precision of the dissection and to improve autonomic nerve identification and preservation, providing better functional outcomes as demonstrated in the nerve-sparing robotic-assisted prostatectomy (94).

## 9 Conclusion

In conclusion, the quality of the available studies on robotic-assisted surgery in deep endometriosis is low despite the encouraging findings on peri-operative outcomes. On the other hand, long-term results about pain relief and pregnancy rates are lacking. We strongly believe that future well-designed studies are required to address these topics and to deeply understand possible advantages of robot-assisted surgery in deep endometriosis. Actually, a prospective randomized controlled single-center trial is ongoing (ROBEndo trial) aiming to evaluate the impact of robotic-assisted surgery for severe deep endometriosis at 6, 12 and 24 months postoperatively and we hope it will help to clarify the role robotic approach.

## Author contributions

FAF: Conceptualization, Writing – original draft. YY: Resources, Writing – review & editing. AN: Resources, Writing – original draft, Writing – review & editing. FF: Writing – review & editing. FO: Writing – review & editing. HK: Supervision, Writing – review & editing. GM: Conceptualization, Methodology, Supervision, Writing – review & editing.
